# Clinical Performance of Paraoxonase-1-Related Variables and Novel Markers of Inflammation in Coronavirus Disease-19. A Machine Learning Approach

**DOI:** 10.3390/antiox10060991

**Published:** 2021-06-21

**Authors:** Elisabet Rodríguez-Tomàs, Simona Iftimie, Helena Castañé, Gerard Baiges-Gaya, Anna Hernández-Aguilera, María González-Viñas, Antoni Castro, Jordi Camps, Jorge Joven

**Affiliations:** 1 Unitat de Recerca Biomèdica, Hospital Universitari de Sant Joan, Institut d’Investigació Sanitària Pere Virgili, Universitat Rovira i Virgili, 43201 Reus, Spain; elisabet.rodriguez@urv.cat (E.R.-T.); helena.castane@urv.cat (H.C.); gerard.baiges@iispv.cat (G.B.-G.); anna.hernandez@urv.cat (A.H.-A.); jorge.joven@urv.cat (J.J.); 2 Department of Internal Medicine, Hospital Universitari de Sant Joan, Institut d’Investigació Sanitària Pere Virgili, Universitat Rovira i Virgili, 43201 Reus, Spain; smiftimie@grupsagessa.com (S.I.); maria.gonzalez@estudiants.urv.cat (M.G.-V.); antoni.castro@urv.cat (A.C.)

**Keywords:** biomarkers, chemokines, COVID-19, galectin-3, machine learning, paraoxonase-1, SARS-CoV-2

## Abstract

SARS-CoV-2 infection produces a response of the innate immune system causing oxidative stress and a strong inflammatory reaction termed ‘cytokine storm’ that is one of the leading causes of death. Paraoxonase-1 (PON1) protects against oxidative stress by hydrolyzing lipoperoxides. Alterations in PON1 activity have been associated with pro-inflammatory mediators such as the chemokine (C-C motif) ligand 2 (CCL2), and the glycoprotein galectin-3. We aimed to investigate the alterations in the circulating levels of PON1, CCL2, and galectin-3 in 126 patients with COVID-19 and their interactions with clinical variables and analytical parameters. A machine learning approach was used to identify predictive markers of the disease. For comparisons, we recruited 45 COVID-19 negative patients and 50 healthy individuals. Our approach identified a synergy between oxidative stress, inflammation, and fibrogenesis in positive patients that is not observed in negative patients. PON1 activity was the parameter with the greatest power to discriminate between patients who were either positive or negative for COVID-19, while their levels of CCL2 and galectin-3 were similar. We suggest that the measurement of serum PON1 activity may be a useful marker for the diagnosis of COVID-19.

## 1. Introduction

Manipulation of host cell function by viral pathogens is vital for successful infection and the creation of a habitat favoring viral replication. The reproduction of viruses depends on the metabolic resources of the host cell for the synthesis of components such as nucleic acids, proteins, and membranes. Most viruses manipulate the host cell’s metabolism in order to optimize the biosynthetic needs of the virus through proviral metabolic changes [1]. Host cells, on the other hand, have developed metabolic strategies to inhibit viral replication through antiviral metabolic changes [2]. Among the mechanisms of innate immunity is the mitochondrial production of mediators that stimulate the transcription of inflammatory cytokines and chemokines, or their maturation by inflammasomes [3,4,5]. Oxidative stress produced by infectious processes may cause mitochondrial dysfunction and this alteration may, in turn, produce a further increase in the production of free radicals [6,7].

The innate immune system has mechanisms that protect against oxidative stress. Among them, paraoxonase-1 (PON1) stands out. PON1 is an enzyme carried into the circulation bound to high-density lipoproteins, which degrades lipoperoxides in lipoproteins and cells [8]. We previously reported that serum PON1 activity was decreased in patients with HIV infection compared to the normal population, and these changes were related to the immunological status of the patients and their degree of inflammation [9]. Other researchers found increased oxidative stress and decreased serum PON1 activities in other viral infections including influenza, hepatitis B, and hepatitis C [10]. Moreover, we and others have reported on the role played in infections by some molecules that participate in the regulation of oxidative stress and inflammatory processes. In this regard, we want to highlight the chemokine (C-C motif) ligand 2 (CCL2), a chemokine that is greatly over-expressed in response to oxidative stress and is responsible for the migration of monocytes to the infection zone and their differentiation into macrophages [11,12,13]. We also want to highlight the glycoprotein galectin-3, which is strongly expressed by macrophages and is a modulator of multiple biological functions, such as proliferation, macrophage chemotaxis, phagocytosis, neutrophil extravasation, neutrophil migration, apoptosis, vacuole lysis after infection, fibrogenesis, and angiogenesis [14,15]. Glycoproteins play a variety of physiological functions, most notably cell-to-cell interactions. In particular, it has been described that galectins 1, 3, and 9 and other glycoproteins, such as syndecans, can promote the internalization of viruses in target cells [15].

COVID-19 is produced by the internalization of the SARS-CoV-2 virus particles in the cells of the respiratory tract after the virus binds to angiotensin-converting enzyme 2 (ACE2), a membrane enzyme responsible for the hydrolysis of the angiotensin II to angiotensin (1-7). The harmful effects of COVID-19 are often linked to an inordinate inflammatory response to a ‘cytokine storm’ which, when uncontrolled, can cause devastating effects on the patient and is the leading cause of a fatal outcome. However, the relationships among PON1, CCL2, and galectin-3 with this strong inflammatory reaction, have not been studied sufficiently.

The present study hypothesizes that SARS-CoV-2 infection can produce alterations in the expression of PON1, CCL2, and galectin-3, which may be involved in the pathophysiology of COVID-19. Thus, our objective was to carry out a comprehensive investigation of the alterations in the circulating levels of these molecules in patients with COVID-19 and the way they interact with the main clinical variables and a wide selection of analytical parameters. We also wanted to identify the best predictive markers, which will be useful in the fight against the effects of the pandemic on our health, our daily lives, and the economy. Our results have been analyzed using classical statistical methods, and also by machine learning, a type of artificial intelligence that is increasingly being developed and used in the interpretation of scientific data.

## 2. Materials and Methods

### 2.1. Participants

This was a post-hoc retrospective cohort study of 126 hospitalized patients with SARS-CoV-2 infection, confirmed by polymerase chain reaction (PCR), recruited between March and October 2020 in the Department of Internal Medicine or the Intensive Care Unit of Hospital Universitari de Sant Joan. The only inclusion criterion was being over 18 years of age. Exclusion criteria were having a life expectancy of less than 24 h, severely altered liver function, or pregnancy. We used two groups of participants as control groups. One group was comprised of 50 healthy volunteers who had participated in an epidemiological study conducted in our geographical area; the details of that study have been reported previously [16]. Those subjects had no clinical or biochemical evidence of renal insufficiency, liver disease, neoplasia, or neurological disorders. The other group was of 45 hospitalized patients, negative for COVID-19, recruited between June and December 2019. Control groups were selected according to a distribution of sexes, ages, and medications, to be as similar as possible to the COVID-19 positive patients. Serum samples from all participants were stored in our Biobank at −80 °C until the time of the study. We recorded clinical and demographic data and calculated the McCabe Score as an index of clinical prognosis [17], and the Charlson Index as a way of categorizing the patients’ comorbidities [18]. This study was approved by the Comitè d’Ètica i Investigació en Medicaments (Institutional Review Board) of Institut d’Investigació Sanitària Pere Virgili (Resolution CEIM 040/2018, amended on 16 April 2020).

### 2.2. Biochemical Analyses

PON1 activity was measured as the rate of hydrolysis of phenylacetate at 280 nm, in a 9 mM Tris-HCl buffer, pH 8.0, and supplemented with 0.9 mM CaCl2, as previously reported. PON1 can hydrolyze multiple substrates, but we chose phenylacetate because it is not toxic, the assay is simple, and is little influenced by *PON1* gene polymorphisms [19]. PON1 concentration was analyzed with the Human PON1 ELISA kit from Elabscience^®^ (Houston, TX, USA). The concentrations of CCL2, interleukin (IL)-10, and IL-6, were analyzed with ABTS ELISA Development kits (Peprotech, London, UK). Galectin-3 was quantified with the Human Galectin-3 Quantikine ELISA Kit (R&D Systems^®^, Minneapolis, MN, USA). Angiotensin II was measured with the Angiotensin II EIA kit from Sigma-Aldrich (St. Louis, MO, USA). ACE2 concentration was analyzed with the DuoSet^®^ ELISA Development System (R&D Systems^®^, Minneapolis, MN, USA). Standard biochemical and hematological analyses were performed in a COBAS^®^ 8000 (Roche Diagnostics, Basel, Switzerland) and a Sysmex XN-1000^TM^ (Sysmex GmbH, Norderstedt, Germany), respectively.

### 2.3. Development of the Predictive Models by Machine Learning

We selected the best predictive model by using the Root Mean Square Error (RMSE) tool, which measures the error of each model by predicting quantitative data. The best model would be the one with the lowest error according to the RMSE. After selection, we trained the models first with 80% of the dataset, and later, we tested the remaining 20% of the dataset. To evaluate the accuracy of each model, we calculated the areas under the curve of the Receiver Operating Characteristics (ROC) curves, and true positive and negative rates were calculated by confusion matrices [20]. The Shapley Additive exPlanation (SHAP) method was used to identify and select the variables with the higher predictive values of each model. This method is a way of determining the contribution (termed SHAP value) of each variable to model outputs. The variables are classified according to their importance. We depicted the SHAP summary plots of the top 20 variables of the chosen prediction model. In plots, the further the value of a variable deviates from zero, the more impact it has on the model output. Positive SHAP values indicate the presence of COVID-19 in patients, while negative values indicate the absence of disease. The colors indicate high (blue) or low (green) levels of each of the variables. The SHAP Partial Dependence Plots were used to identify the cut-offs of the variables selected by SHAP that better discriminate the presence of COVID-19. Scikit-learn package [21,22] in Python was used to implement machine learning models, SHAP values, and the Partial Dependence Plot.

### 2.4. Statistical Analyses

All statistical calculations and graph representations were made using the Statistical Package for Social Sciences (SPSS 24.0, Chicago, IL, USA) and GraphPad Prism 6.01 (GraphPad Software, San Diego, CA, USA). Since most of the studied variables had non-Gaussian distributions, differences between any two groups were assessed by the Mann-Whitney *U* test. Qualitative data were analyzed using the χ^2^ test. R statistics was employed to calculate the Spearman pairwise correlation matrices through the ggcorrplot package [23].

## 3. Results

### 3.1. Relationships between PON1-Related Variables and Novel Inflammation Markers with the Clinical Characteristics of the Study Groups

**Figure 1:**
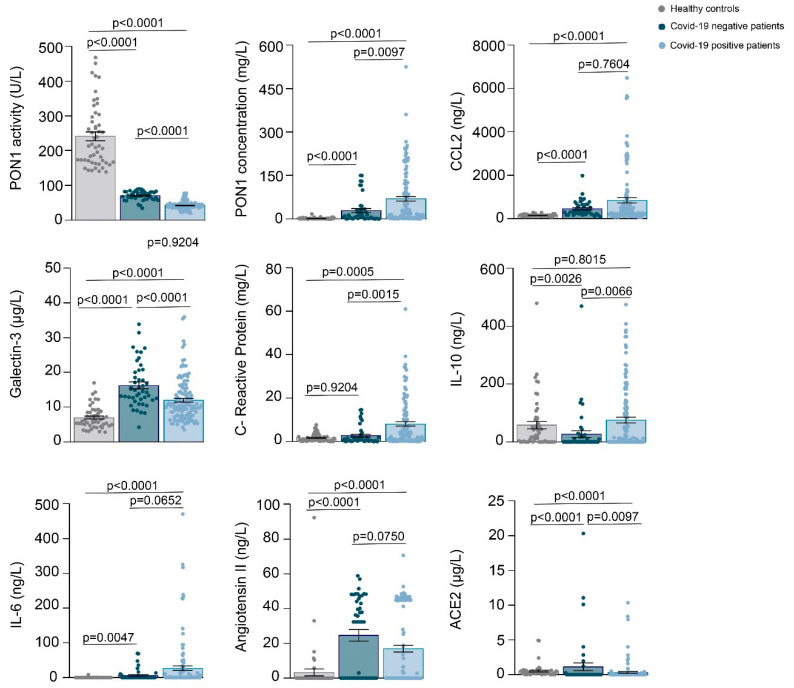
Scatter plots with error bars of selected variables in healthy controls, patients COVID-19 negative and COVID-19 positive. Results are given as means and standard error of the mean (SEM). Statistical analyses were performed using the Mann-Whitney *U* test.

**Figure 2:**
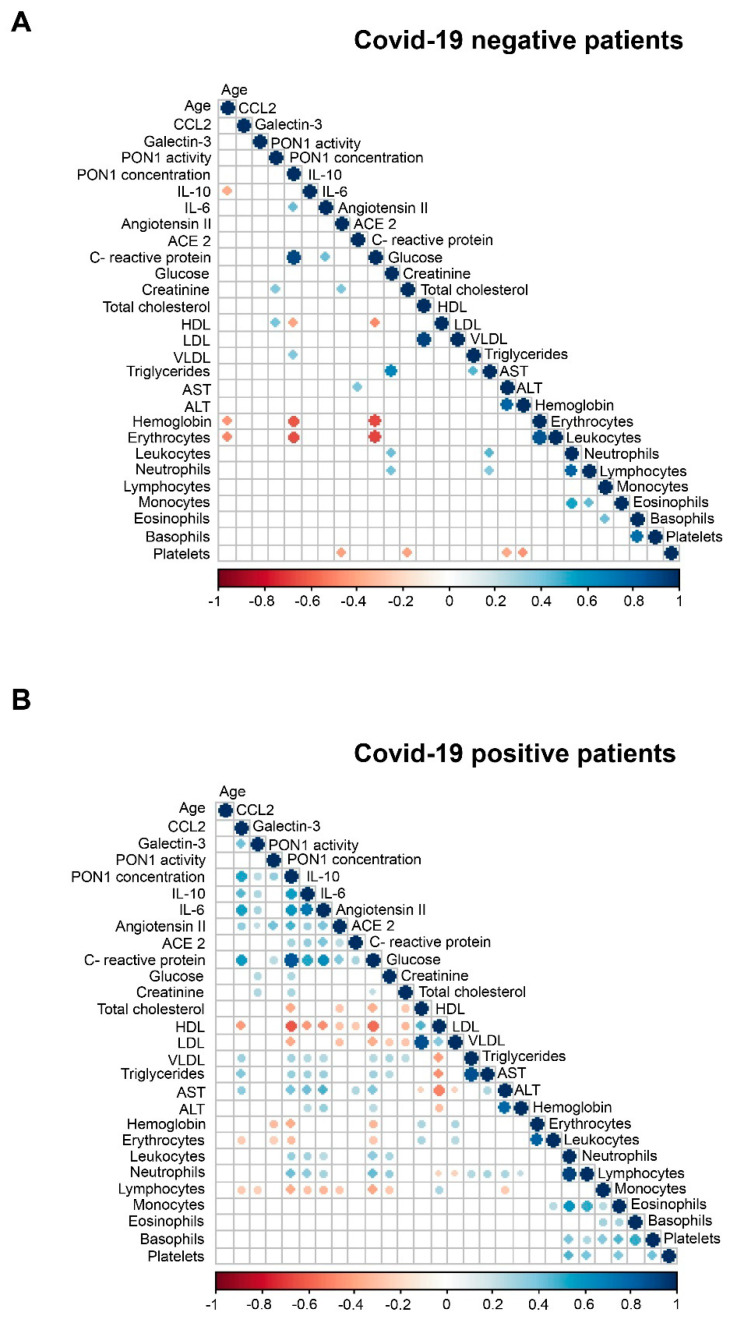
Spearman correlation matrices of analytical variables in COVID-19 negative (**A**) and COVID-19 positive (**B**) patients. The magnitude and direction of the correlations are shown by circle size (larger is stronger) and with colors (positive correlation: blue; negative correlation: red), respectively.

The demographic and clinical characteristics of all participants are given in Table 1. Patients negative for COVID-19 were significantly older and had a lower frequency of alcohol intake than the healthy individuals. Patients positive for COVID-19 had a lower frequency of smoking habits, alcohol intake, type 2 diabetes mellitus, chronic kidney disease, and cancer than COVID-19 negative patients. The McCabe Score and the Charlson Index indicated that COVID-19 positive patients were, in general, less severe than negative patients. Results of the selected biochemical variables are given in Figure 1. Both groups of hospitalized patients had significantly lower PON1 activities and higher PON1, CCL2, galectin-3, CRP, IL-6, angiotensin II, and ACE2 concentrations than the healthy subjects. COVID-19 positive patients had lower PON1 activities and galectin-3 concentrations, and higher PON1, CRP, IL-10, and ACE2 concentrations than COVID-19 negative patients. Figure 2 summarizes the correlations between all the analyzed parameters. Considering the objectives of our study, we highlight that there were significant, direct correlations between CCl2, galectin-3, PON1 concentration, CRP, IL-10, IL-6, and angiotensin II in COVID-19 positive patients, but not in the COVID negative ones.

**Figure 3:**
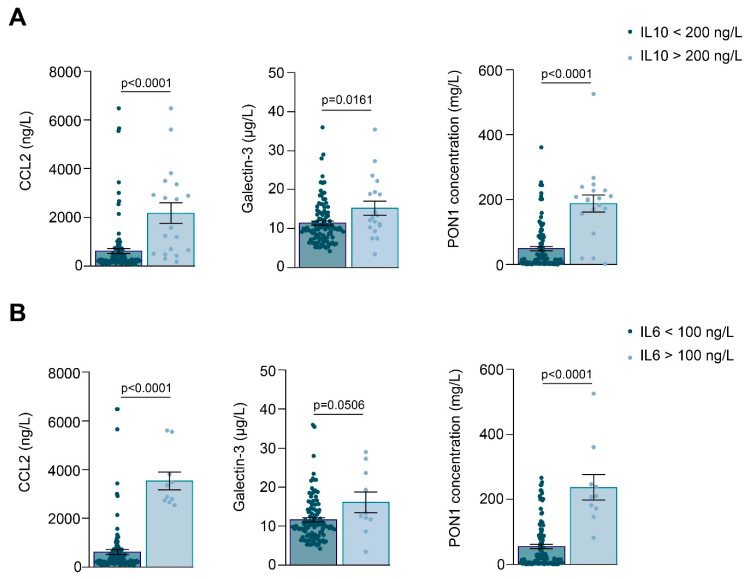
Interleukin (IL)-10 (**A**) and IL-6 (**B**) concentrations are associated with CCL2, galectin-3, and PON1 concentrations in COVID-19 positive patients. The figure shows scatter plots with error bars of selected variables segregated according to IL-10 and IL-6 concentrations. Results are given as means and standard error of the mean (SEM). Statistical analyses were performed using the Mann-Whitney *U* test.

**Figure 4:**
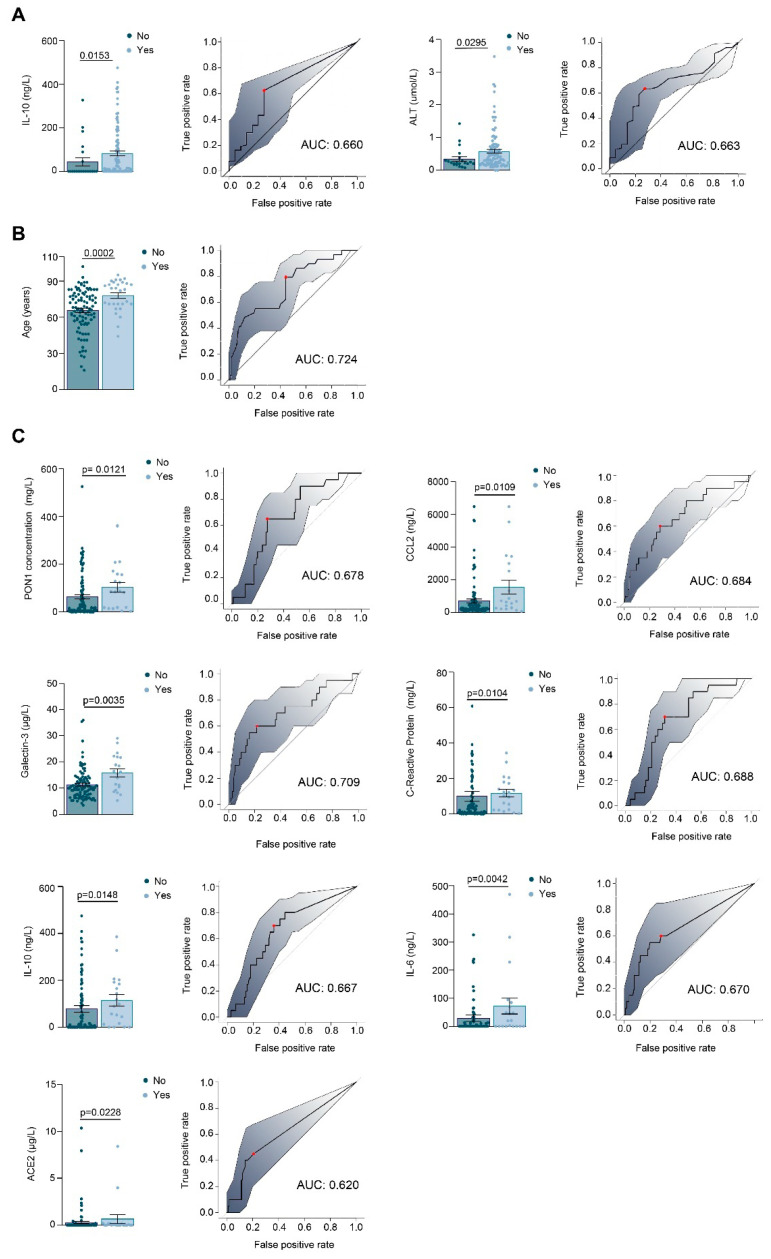
Relationships between selected variables and the clinical characteristics of COVID-19 positive patients. The figure shows scatter plots with error bars and Receiver Operating Characteristics curves of the variables that presented with significant differences in relation to whether patients were admitted to Intensive Care Unit (**A**), died during their hospital stay (**B**), or received invasive mechanical ventilation (**C**). Results are given as means and standard error of the mean (SEM). Statistical analyses were performed using the Mann-Whitney *U* test.

Considering only the COVID-19 positive patients, we found that those with higher concentrations of IL-10 (>200 ng/L) or IL-6 (>100 ng/L) had higher concentrations of CCL2, galectin-3, and PON1 concentrations (Figure 3). The number of COVID-19 positive patients admitted to the Intensive Care Unit, the respiratory interventions carried out, and the number of deaths are given in Table 2. Patients in the ICU had higher IL-10 concentrations and ALT activities, and those treated with an invasive mechanical intervention had higher concentrations of PON1, CCL2, galectin-3, CRP, IL-10, IL-6, and ACE2. However, despite these differences, the areas under the curve of the receiver operating characteristics plots were relatively low, indicating that none of these parameters alone is sufficient to discriminate between the presence or absence of each of the clinical characteristics studied. (Figure 4). We did not find any significant differences between the parameters investigated in the patients who died, who were exclusively characterized by older age. We did not find any other relationship between PON1-related variables and novel inflammation markers with any other clinical characteristics or comorbidities of COVID-19 positive patients, including type 2 diabetes mellitus, cancer, and chronic kidney disease.

### 3.2. Machine Learning Identified Serum PON1 Activity as the Best Analytical Parameter to Discriminate between COVID-19 Positive and Negative Patients

**Figure 5:**
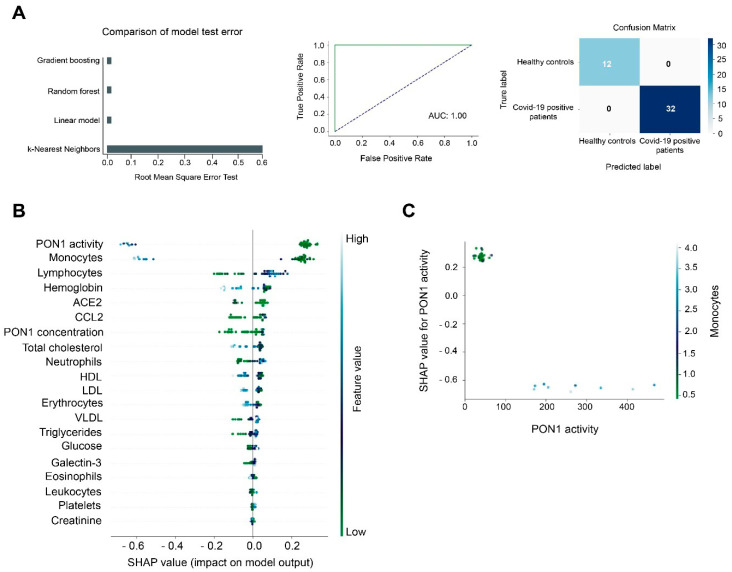
Gradient Boosting Machine (GBM) as a diagnostic model of prediction of COVID-19. PON1 activity and monocyte concentrations discriminate between COVID-19 positive patients and the healthy population. (**A**) Root mean squared error test of the GBM, Random Forest, Lineal Model, and K-Nearest Neighbors. The Receiver Operating Characteristic curve and matrix confusion of the GBM model shows an area under the curve (AUC) of 1.00. (**B**) A SHapley Additive exPlanations (SHAP) summary plot of the GBM shows the top 20 features predicting COVID-19. Positive SHAP values indicate the presence of COVID-19 in patients, while negative values indicate the absence of disease. The colors indicate high (light blue) or low (light green) levels of each of the variables. SHAP values on the *x*-axis indicate the distribution of the prediction among the features. (**C**) The relationship between PON1 activity and monocyte concentration is shown by the Partial Dependence plot.

If conventional statistics allow us to evaluate the differences of each individual parameter between the different study groups, machine learning models allow us to make predictions by analyzing all the investigated parameters globally. According to RMSE, Gradient Boosting Machine (GBM) was the best model to make predictions when comparing COVID-19 positive patients with healthy controls. We trained the model with 80% of our dataset and later we tested the model using the remaining 20% of the dataset. The test model presented an ROC plot area under the curve (AUC) of 1.00 and the matrix confusion showed 0% of false positive and false negative rates (Figure 5A). The SHAP summary plot revealed that PON1 activity and monocyte concentrations were the best variables for discriminating between both study groups (Figure 5B). The lower the monocyte concentrations and the PON1 activity, the more likely a patient was to be COVID-19 positive. We observed an inverse prediction (SHAP value of −0.6) to be COVID-19 positive at higher PON1 activity and monocyte concentrations. Indeed, when PON1 activity was higher than 100 U/L, and monocyte concentrations were higher than 2 × 10^9^/L, the model predicted an absence of COVID-19 (Figure 5C).

**Figure 6:**
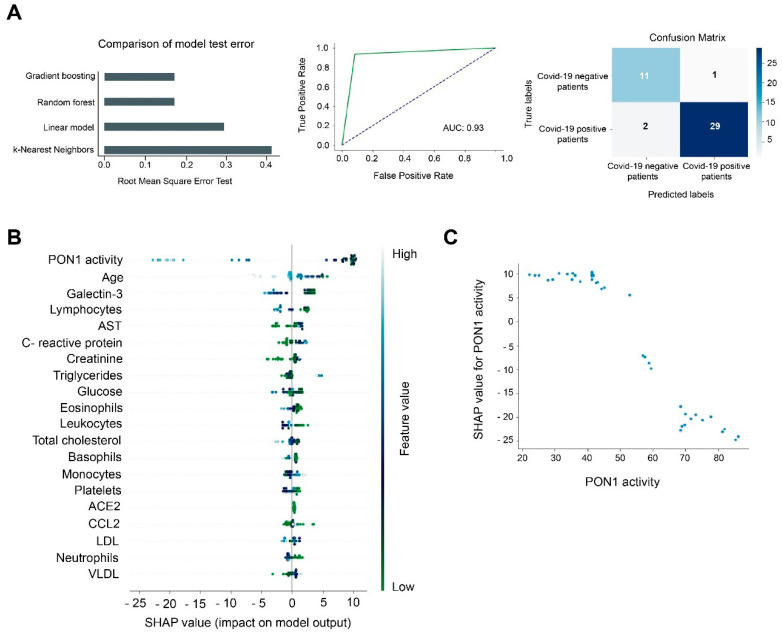
Gradient Boosting Machine (GBM) as a diagnostic model of prediction of COVID-19. PON1 activity discriminates between COVID-19 positive and negative patients. (**A**) Root mean squared error test of the GBM, Random Forest, Lineal Model, and K-Nearest Neighbors. The Receiver Operating Characteristic curve and matrix confusion of the GBM model shows an area under the curve (AUC) of 0.93. (**B**) SHapley Additive exPlanations (SHAP) summary plot of the GBM showing the top 20 features predicting COVID-19. Positive SHAP values indicate the presence of COVID-19 in patients, while negative values indicate the absence of disease. The colors indicate high (light blue) or low (light green) levels of each of the variables. SHAP values on the *x*-axis indicate the distribution of the prediction among the features. (**C**) Partial Dependence plot of PON1 activity.

When we compared hospitalized COVID-19 positive vs. COVID-19 negative patients, GBM was again the best model, with an AUC of 0.93 and with lower rates of false positives (1 case) and false negatives (2 cases) (Figure 6A). PON1 activity was the best feature for discriminating between these groups (Figure 6B). Higher PON1 activity was associated with a lower probability of being COVID-19 positive. By contrast, when this activity decreased, the prediction of being COVID-19 increased. The Partial Dependence plot illustrated that those patients with an activity of 70 U/L or more were unlikely to be COVID-19 positive, but when the activity was below 50 U/L, the probability of being COVID-19 positive was high (Figure 6C).

## 4. Discussion

The present study found a marked decrease in PON1 activity and an increase in PON1, CCl2, and galectin-3 concentrations in COVID-19 positive hospitalized patients, compared to healthy individuals. When comparing COVID-19 positive with COVID-19 negative patients, we also found higher PON1 concentrations in the former, but no significant differences were observed with respect to CCL2, and galectin-3 concentrations were lower. We think that the most remarkable result of our study is the sharp reduction in PON1 activity in COVID-19 positive patients, which seems specific to this infectious disease. This reduction is significant with respect to COVID-19 negative patients, despite their being of more advanced age and having a more serious pathological history and comorbidities, as measured by the Charlson and McCabe indices. Data on the alterations in PON1 levels in COVID-19 is scarce, but recent studies show that it may be important. The little data existing seems to suggest two different roles for PON1 in COVID-19, depending on whether it is present in the circulation or within cells. Purified native HDL with intact PON1 elicits a potent antiviral effect against SARS-CoV-2 in cultured monocyte cells, while glycated HDL, with inactive PON1, loses the antiviral activity [24]. However, an in silico study reported that PON1 enhances the action of ACE2, the main cell receptor of SARS-CoV-2 [25], and the inhibition of PON1 activity has been reported as a potent inhibitor of vaccinia virus protein synthesis and viral mRNA methylation in mice [26], suggesting that intracellular PON1 is important in the translation of viral proteins and virus replication.

PON1 activity in the serum of COVID-19 patients may be lower for several, though not mutually exclusive, reasons. SARS-CoV-2 infected patients are known to often have low HDL levels [27], and recent studies reported that patients with severe COVID-19 had lower HDL cholesterol and higher triglyceride levels before and after the infection than patients without severe COVID-19 or the healthy population [28,29]. Begue et al. [29] found that the HDL cholesterol concentration of COVID-19 patients admitted to the Intensive Care Unit was about half that of healthy individuals and that their HDL particles were enriched in various inflammatory proteins and depleted in PON1. However, the decrease in HDL concentrations alone cannot justify the dramatic decrease in PON1 activities. The increase in oxidative stress secondary to infection is likely the most important reason for the large decrease in PON1 activity. This enzyme is a lipoperoxide hydrolase and, to exert its action, the active center of the enzyme has to bind covalently to the substrate molecules, the result being that the enzyme is inactivated [8]. This occurs despite an increase in its serum concentration. We have already observed changes in the opposite direction of the activity and concentration of PON1 in other infectious and non-infectious diseases that involve oxidative stress, and we interpret them as an attempt by the organism to counteract the decrease in enzyme activity [9,12,13,30,31,32,33,34]. On the other side, we measured the concentration of PON1 in serum by ELISA, and therefore, we cannot be sure if the PON1 protein was all located in the HDL, or was partially displaced to the LDL or to the circulation, which would contribute to the decrease in its activity.

Several studies have shown that reduced PON1 activity is associated with an enhanced inflammatory response, CCL2 synthesis, and secretion [35,36,37]. High levels of this chemokine have been found in the circulation [38,39,40,41,42,43], the bronchoalveolar lavage fluid [44], and the lung tissue of COVID-19 patients [45]. One study reported that CCL2 expression increases rapidly in the early acute phase of infection and then progressively decreases as the disease advances [46]. Our results also show higher serum CCL2 concentrations, however, their contribution is new, which seems to suggest that there are two subpopulations depending on the levels of CCL2. One presents a moderate increase, similar to that of negative COVID-19 hospitalized patients, and another presents very high CCL2 levels—more than 20 times higher than those of healthy individuals. We have not been able to find any explanation for these differences. Indeed, we have not found any association between the levels of CCL2 and the clinical characteristics of the patients and comorbidities. The only association that we have found in this regard is that the majority of patients with very high levels of CCL2 (but not all) needed to receive invasive mechanical ventilation in the Intensive Care Unit. This observation could be explained by the combined effect of the greater severity of these patients together with an inflammatory reaction merely produced by the invasive intervention [47].

We also found higher serum galectin-3 concentrations in COVID-19 positive patients, which were even higher in those receiving invasive mechanical ventilation. Galectin-3 facilitates viral infection. In HIV infection, it serves as an attachment factor that favors viral entry into T-cells [48]. HIV infection also increases galectin-3 expression through activation of NF-kB–dependent pathways, and secreted galectin-3 then induces an increase in pro-inflammatory cytokine synthesis, including tumor necrosis factor, IL-1β, and IL-6, among others [49]. Patients with severe COVID-19 showed elevated levels of galectin-3, tumor necrosis factor, IL-1, and IL6, compared to those with moderate disease or normal subjects [48,50,51]. Our results confirm that COVID-19 positive patients have higher serum galectin-3 concentrations than normal individuals. However, an interesting finding is that galectine-3 levels in COVID-19 positive patients were significantly lower than those of the hospitalized COVID-19 negative patients. The explanation for this observation cannot be inferred from our results. Indeed, there is no reason for us to think *a priori* that they should be higher. There is a possibility that the ELISA method underestimates galectin-3 concentrations in COVID-19 positive patients, which could be because the SARS-CoV-2 protein S contains a domain that is practically homologous to this lectin [52]. Thus, virus particles perhaps compete with galectin-3 for antibody binding sites in the ELISA, resulting in artificially low results.

We found significant direct correlations between galectin-3, CCL2, PON1, IL-6, IL-10, CRP, ACE2, and angiotensin II concentrations in COVID-19 positive patients as well as with aminotransferase activities and triglycerides, and inverse correlations with HDL-cholesterol. Patients with the highest interleukin levels also had higher PON1, CCL2, and galectin-3 concentrations. These results suggest a synergy between oxidative stress, inflammation, and fibrogenesis in positive patients that is not observed in negative patients, and therefore appears to be a specific characteristic of COVID-19. To analyze the many analytical and clinical variables and their interrelationships together, we used a machine learning approach. Machine learning is an efficient and reliable tool for studying and integrating a large amount of data. In our case, it has allowed us to create predictive models that define the differences between positive COVID-19 patients and healthy individuals, and between positive and negative COVID-19 patients. This analysis identified PON1 activity as the individual parameter that best discriminated these patients. When positive patients were compared with healthy individuals, the SHAP analysis identified PON1 activity and monocyte levels as the parameters with the highest discriminatory power, but the differences in monocytes were not maintained when the positive and negative patients were compared. These results suggest that the determination of PON1 activity may be consistently useful for the diagnosis of COVID-19, but the concentration of monocytes may not be so in individuals with chronic diseases other than COVID-19.

Our results suggest that the determination of PON1 activity in serum may be a useful marker for the diagnosis of COVID-19. According to the SHAP and Partial Dependence Plots, their greatest use could be to rule out disease, which would also be of great clinical interest. This analysis is fast, it does not last more than 5 min and, given its simplicity, it can be easily adaptable to any automated analyzer with open channels. Studies currently undergoing in our laboratory are validating the usefulness of the automated method in a large prospective series of outpatients and hospitalized patients with different levels of severity of COVID-19.

## 5. Conclusions

Taken together, the results from the present study highlight that serious metabolic alterations are a consequence of SARS-CoV-2 infection and show how the study of these alterations can help us understand the molecular basis of this infection and identify new markers of the disease with obvious clinical uses.

## Figures and Tables

**Table 1:** Demographic and clinical characteristics of the patients and the healthy subjects.

		Healthy Subjects *n* = 50	COVID-19 Negative Patients *n* = 45	COVID-19 Positive Patients *n* = 126
**Demographic variables**				
Sex, male		38 (76.0)	30 (66.7)	68 (54.8) ^a^
Age, years		75 (66–84)	84 (75–89) ^b^	71 (58–83)
Smoking, n (%)		19 (38.0)	16 (35.6)	6 (4.8) ^b,d^
Alcohol intake, n (%)		28 (56.0)	7 (15.5) ^b^	6 (4.8) ^b,c^
**Comorbidities**				
Type 2 diabetes mellitus, n (%)		0	22 (48.9)	30 (23.8) ^e^
Cardiovascular disease, n (%)		0	18 (40)	68 (54)
Chronic liver disease, n (%)		0	0	1 (0.8)
Chronic lung disease, n (%)		0	0	18 (14.3)
Chronic kidney disease, n (%)		0	19 (42.2)	22 (17.5) ^d^
Chronic neurological disease n (%),		0	0	29 (23)
Cancer, n (%)		0	17 (37.8)	16 (12.7) ^e^
Charlson Index	No comorbidity, n (%)	NA	10 (22.2)	83 (65.9) ^e,^*
	Low comorbidity, n (%)		18 (40.0)	29 (23.0)
	High comorbidity, n (%)		17 (37.8)	14 (11.1)
McCabe Index	RFD, n (%)	NA	10 (22.2)	7 (5.6) ^e,^ *
	UFD, n (%)		19 (42.2)	31 (24.6)
	NFD, n (%)		16 (35.6)	88 (69.8)
**Medications**				
ACEIs, n (%)		NA	14 (31.1)	24 (27.0)
ARAs, n (%)		NA	12 (26.7)	21 (16.7)
Oral antidiabetics, n (%)		NA	19 (42.2)	37 (29.4)
Insulin, n (%)		NA	9 (20.0)	28 (22.2)
Statins, n (%)		NA	16 (35.6)	44 (34.9)

^a^*p* < 0.01, ^b^ *p* < 0.001, with respect to healthy subjects; ^c^ *p* < 0.05, ^d^ *p* < 0.01, ^e^ *p* < 0.001 with respect to COVID-19 negative patients. *: Global P-value including the three categories of each index. Statistical analyses were performed by the Student’s *t-test* (quantitative) or the χ-square test (qualitative). Results are given as medians and 95% CI, or as numbers and percentages. ACEIs: Angiotensin-converting enzyme inhibitors; ARAs, Angiotensin II receptor antagonists; NFD: Non-fatal disease; RFD: Rapidly fatal disease. UFD: Ultimately fatal disease.

**Table 2:** Selected clinical variables in COVID-19 positive patients.

Variable	Number of Cases (%)
Admission to Intensive Care Unit	22 (17.5)
Non-invasive mechanical ventilation	6 (4.8)
Invasive mechanical ventilation	20 (15.9)
High-flow oxygen therapy	10 (7.9)
Conventional oxygen therapy	92 (73.0)
Deceased	29 (23.0)

## Data Availability

The data presented in this study are available on request from the corresponding author.
